# Toxic Metal and Essential Element Concentrations in the Blood and Tissues of Pancreatic Ductal Adenocarcinoma Patients

**DOI:** 10.3390/toxics12010032

**Published:** 2024-01-01

**Authors:** Giovanni Forte, Andrea Pisano, Beatrice Bocca, Grazia Fenu, Cristiano Farace, Federica Etzi, Teresa Perra, Angela Sabalic, Alberto Porcu, Roberto Madeddu

**Affiliations:** 1 Department of Environment and Health, Italian National Institute of Health, 00161 Rome, Italy; giovanni.forte@iss.it; 2 Department of Biomedical Science—Histology, University of Sassari, 07100 Sassari, Italy; pisanoandrea87@gmail.com (A.P.); grazia.fenu94@gmail.com (G.F.); cfarace@uniss.it (C.F.); f.etzi@studenti.uniss.it (F.E.); angelasabalic08@gmail.com (A.S.); rmadeddu@uniss.it (R.M.); 3 National Institute of Biostructures and Biosystems, Interuniversity Consortium INBB, 00136 Rome, Italy; 4 Department of Medicine, Surgery and Pharmacy, Unit of General Surgery, University of Sassari, 07100 Sassari, Italy; teresaperra1992@gmail.com (T.P.); alberto@uniss.it (A.P.)

**Keywords:** toxic metals, essential elements, pancreatic ductal adenocarcinoma, whole blood, pancreatic tissue

## Abstract

Background: Pancreatic ductal adenocarcinoma (PDAC) is a highly aggressive lethal neoplasm, and it has an average 5-year survival rate of less than 10%. Although the factors that influence PDAC development remain unclear, exposure to toxic metals or the imbalance in essential elements may have a role in PDAC-associated metabolic pathways. Methods: This study determined the concentrations of Cd, Cr, Cu, Fe, Mn, Ni, Pb, Se and Zn in whole blood, cancer and non-cancer tissues of patients affected by PDAC, and compared them with levels in healthy controls using inductively coupled plasma mass spectrometry. Results: Results of the whole blood showed significantly higher levels of Cr, Cu and Cu/Zn ratio in PDAC patients compared to the controls. In addition, the concentrations of Cu, Se, Fe and Zn significantly increased in cancer tissue compared to the healthy counterparts. Conclusions: This study revealed evidence of altered metal levels in the blood and pancreatic tissues of PDAC patients with respect to healthy controls. These changes may contribute to multiple mechanisms involved in metal-induced carcinogenesis, including oxidative stress, DNA damage, genetic alteration, decreased antioxidant barriers and inflammatory responses. Thus, the analysis of metals can be used in the diagnosis and monitoring of PDAC neoplasms.

## 1. Introduction

The pancreas produces some important hormones including insulin and glucagon able to maintain healthy blood sugar levels. Moreover, the pancreas secretes various enzymes, such as trypsin, lipase and amylase that contribute to the proper digestion and absorption of fats, proteins and carbohydrates. About 70% of pancreatic cancers develop in the head of the organ, and most of them originate in ducts that carry digestive enzymes. Having an insufficient amount of pancreatic enzymes is very common among people with pancreatic cancer. Pancreatic ductal adenocarcinoma (PDAC) is the most common histologic type of pancreatic cancer and is one of the most aggressive malignant neoplasms with poor outcomes, and subjects most at risk are those in the age range between 50 and 80 years [1]. According to the WHO, in 2019, deaths in Italy due to PADC amounted to ca. 13,000, the age standardized death rate was 7.9%, the death rate for 100,000 subjects was 21.6% and the percentage of cause-specific deaths out of total deaths was 2% [2]. The onset and increase in PADC numbers have a multifactorial origin. In 10% of cases, there is a genetic basis with mutations of different genes, while in remaining cases, cigarette smoke, alcohol, incorrect diet, diabetes, obesity and environmental pollutants have been associated with an increased risk of PDAC neoplasms [1].

Among environmental pollutants, exposure to toxic metals such as Cd, Cr, Ni and Pb may have an impact in PDAC neoplasms. In particular, a study explored the effects of Cd on three different lines of evidence: a case control study on patients with pancreatic cancer; an animal study employing Cd-treated Wistar rats; and an in vitro study using Cd-exposed pancreatic cells such as hTERT-HPNE (human pancreatic Nestin-expressing cells) and AsPC-1 (human pancreatic tumor cell line). The results indicated Cd accumulation in human and rat pancreatic tissues, disturbances in the intrinsic pathway of apoptotic activity and oxidative stress elevation in pancreatic cells [3]. Moreover, Cd was a competitor for pancreatic Zn due to its similar physico-chemical properties. The same authors also reported that Cd acted in a mitogenic manner to pancreatic cells and was able to cause their transdifferentiation and increase both the synthesis of pancreatic DNA and oncogene activation [4].

Regarding Cr and Ni, the literature supports the concept that these metals may interfere with key steps in repairing DNA damage and they may also stimulate cell proliferation via the activation of early response genes or interference with genes down-regulating cell growth and senescence [5,6]. A study reported significantly higher levels of Ni in pancreatic cancerous tissues with respect to the controls [7].

Many microRNAs (miRNAs) have an essential role in the regulation of oncogenes or tumor suppressor genes in cell signaling pathways, and the dysregulation of miRNAs is a hallmark of cancer. In vitro studies found high expression levels of two miRNAs, miR-221 and miR-155, in pancreatic tumor cell lines exposed to increasing Ni concentrations, while the expression level of miR-126 was significantly decreased [7].

Authors investigated the relationship between toenail concentrations of trace elements and occupational history in pancreatic cancer patients. [8]. They observed that patients exposed to aromatic hydrocarbon solvents presented high levels of metals such as Cd, Mn, Pb and Fe, whilst patients exposed to pesticides showed increased Cd and Mn values, and patients exposed to formaldehyde and chlorinated hydrocarbon solvents reported higher levels of Pb [8].

Moreover, despite metals such as Cu, Fe, Mn, Se and Zn being essential for normal biological functioning [9,10], the alteration in their levels may increase cellular oxidative stress, posing the basis for the development of diseases. Regarding Cu and Se, both micronutrients are involved in many biochemical processes, including cellular respiration, cellular utilization of oxygen, DNA and RNA production, maintenance of cell membrane integrity and sequestration of free radicals. Copper is important for functions involved in cell proliferation or angiogenesis, which are central to tumorigenesis and cancer development [11]. For Se, a higher intake of Se might reduce the risk of pancreatic cancer [12]. Iron is an essential element for life due to its role in synthesizing oxygen transport proteins such as hemoglobin, myoglobin and other Fe-containing proteins. It is widely acknowledged that an excess accumulation of Fe can be harmful by increasing the production of reactive oxygen species (ROS), which ultimately cause DNA damage and genetic alterations, leading to the basis of pancreatic cancer growth [13]. Other authors reported on the role of Mn in carcinogenesis via the generation of oxygen radicals, suppression of apoptosis and binding competition between chromatin and metal ions in molecules [14]. Zinc is involved in a multitude of processes within the pancreas, including glucagon secretion, digestive enzyme activity and insulin packaging, secretion and signaling. A dysregulation of Zn metabolism within the pancreas impaired a multitude of key processes, including glycemic control, pancreatic cancer and chronic pancreatitis [15].

Literature data suggested that multiple Zn-regulated transporters (Zip) play a role in the intracellular concentrations of Zn during the development and progression of pancreatic cancer. There is evidence of the overexpression of Zrt-Irt-like protein 4 (Zip4) in pancreatic cancers allowing for Zn accumulation [16], whilst other studies reported a downregulation of Zip8 and Zip3 transporters in adenocarcinoma tissue sections accompanied by the loss of intracellular Zn during well-differentiated and progressing pancreatic malignancy [17].

Building upon the aforementioned background, increased levels of toxic metals in the body and variations in the concentrations of essential elements might be associated with the development of pancreatic cancer. To this end, the present study determined Cd, Cr, Cu, Fe, Mn, Ni, Pb, Se and Zn in the whole blood and cancer tissues of patients affected by PDAC with respect to the levels of these elements in healthy controls. The final objective was to investigate the use of metals for the diagnosis and monitoring of PDAC neoplasms.

## 2. Materials and Methods

### 2.1. Characteristics of the Subjects

Forty-six subjects affected by PDAC (mean age, 67.2 ± 9.4 years; 21 males and 25 females) and 20 healthy controls (HC; mean age, 60.5 ± 8.5 years; 11 females and 9 males) were enrolled in this study, and their details are reported in Table 1. Whole blood samples were collected in EDTA tubes for trace metals (BD Vacutainer, Franklin Lakes, NJ, USA) from all 46 PDAC patients, including 32 operated PDAC patients (OPPs) and 14 non-operated PDAC patients (NOPPs) that corresponded to cancer stage IV, and 20 HCs. Furthermore, PDAC tissues and adjacent non-cancer tissue samples were collected from the 32 OPPs. PDAC patients were also subdivided according to the severity of the disease as per the American Joint Committee on Cancer (AJCC) pancreatic cancer stages [18]. In particular:

- Stage 0 (carcinoma in situ): cancer cells are only present in the top layers of pancreatic duct cells and have not invaded deeper tissues;
- Stage Ia: cancer (max. 2 cm) is confined to the pancreas and has not spread beyond;
- Stage Ib: cancer (larger than 2 cm) is confined to the pancreas and has not spread beyond;
- Stage IIb: cancer has extended beyond the pancreas (from 1 to 3 regional lymph nodes) but has not invaded major blood vessels;
- Stage III: cancer has spread to nearby major blood vessels but has not spread to distant organs;
- Stage IV: cancer has spread to distant organs, such as the liver, lungs or other organs.

The numbers of patients within each stage are reported in Table 1.

All subjects were natives of Sardinia and had the same ethnic origin. Age and gender-matched controls were blood donors with no history of cancer, and resided in the same geographical area of the patients. The study protocol was approved by the Institutional Ethical Committee of the University of Cagliari (protocol number PG/2021/8575) and the Health Directorate of Sassari (protocol number 464), and informed written consent was obtained from each subject. A questionnaire was administered to all individuals (cases and controls) and relevant personal data (e.g., gender, age, smoke, alcohol, metallic prosthesis, occupational exposure to metals) were collected. All subjects were non-smokers and non-drinkers, none of the subjects were occupationally exposed to metals and none of them had metallic prosthesis in the body. The study was conducted according to the declaration of Helsinki.

### 2.2. Sample Preparation

One mL of whole blood from each subject was collected into a 15 mL polystyrene tube (Corning, Glendale, AZ, USA), and was added to 2 mL of ultrapure HNO_3_ (VWR, Leuven, Belgium) and digested on a heat block (ModBlock, CPI International, Santa Rosa, CA, USA) at 80 °C until complete dissolution. The digests were further diluted with ultrapure deionized water (Micro Pure UV, Thermo Scientific Barnstead, Langenselbold, Germany).

Approximately 0.150 g of cancer and non-cancer tissues were dried at 105 °C overnight (the water content was ca. 50%). Further, samples were weighed in 15 mL polystyrene tubes, added with 2 mL of ultrapure HNO_3_ (VWR), digested on a heat block at 80 °C until complete dissolution (ModBlock), and then diluted with ultrapure, deionized water for analysis.

### 2.3. Quantification of Metals

Thermo Scientific iCAP Qc inductively coupled plasma mass spectrometry (ICP-MS) (Bremen, Germany) was used to quantify the following metals: ^114^Cd, ^52^Cr, ^63^Cu, ^56^Fe, ^55^Mn, ^60^Ni, ^82^Se, ^208^Pb and ^64^Zn. In order to efficiently reduce polyatomic interferences in analytical masses, metals were quantified using the He-pressurized QCell in the kinetic energy discrimination (KED) mode. Procedural blanks to assess the possible exogenous metal contamination from plastics and reagents were analyzed. The addition calibration method was used to quantify the elements, and ^103^Rh at 1 ng/mL in the analytical solutions was used as an internal standard to account for possible instrumental drifts. The limits of detection (LoD) was calculated as three times the standard deviation of replicated measurements of the pooled digested samples (blood or tissue). Analytical details and ICP-MS settings are reported in Supplementary Materials and Table S1.

In whole blood analysis, certified reference material (CRM) ClinChek^®^ Level I Whole Blood Control (Recipe, Munich, Germany) was analyzed to determine the recovery method and intra-day precision (Table S2 in Supplementary Materials). Recovery was in the range of 90–110% and intra-day precision between 3.1 and 7.5% for all elements. With reference to tissue analysis, CRM pig kidney ERM–BB186 and muscle tissue ERM-CE278k (IRMM, Geel, Belgium) were analyzed (Tables S3 and S4, respectively, in Supplementary Materials). For all elements, the recovery was between 94 and 117%, and intra-day precision ranged between 4.7 and 7.5%.

### 2.4. Statistics

Since data were not normally distributed, the results were expressed as the median (50th percentile) and 5th–95th percentiles. Differences between the metal levels in PDAC patients and controls, and metal–variables associations (sex, age, and stage of disease) were tested using non-parametric tests (U Mann–Whitney, Kruskal–Wallis, Spearman’s ρ, Wilcoxson for coupled tissue samples) and *p*-values of <0.05 were considered statistically significant. Moreover, receiver operating characteristic (ROC) curves were used to establish a concentration threshold value in blood and cancer tissue able to distinguish the PDAC patients and healthy controls. The quality of ROC results was expressed in terms of the area under the ROC curve (AUC), specificity (proportion of correctly identified healthy controls, also referred to as the false positive rate) and sensitivity (proportion of correctly identified PDAC patients, also called the true positive rate). IBM SPSS Statistics 28 was used as the statistical package.

## 3. Results

Table 2 reports on the concentrations of toxic metals and essential elements in the whole blood of PDAC patients, OPPs, NOPPs and the HC group. The results of the blood showed significantly higher levels of Cr and Cu (both *p* < 0.01) in PDAC patients when compared to the HCs. Similarly, significantly higher concentrations of Cr and Cu were observed in OPPs (Cr, *p* < 0.03; Cu, *p* < 0.01) and NOPPs (Cr and Cu, both *p* < 0.03) with respect to the HCs.

When considering gender, the levels of blood Pb were increased in males compared to females in all groups, i.e., PDAC patients (median; 43.4 ng/mL vs. 17.2 ng/mL; *p* < 0.01), OPPs (median; 44.7 ng/mL vs. 17.7 ng/mL; *p* < 0.01) and NOPPs (median; 40.1 ng/mL vs. 16.5 ng/mL; *p* < 0.03).

In PDAC females, the concentrations of blood Cu were higher than in HC females (median; 1269 ng/mL vs. 982 ng/mL; *p* < 0.03). In PDAC males, levels of blood Cu (median; 1202 ng/mL vs. 858 ng/mL; *p* < 0.05) and Pb (median; 43.3 ng/mL vs. 17.9 ng/mL; *p* < 0.01) increased respect to HC males. On the contrary, in PDAC males, the levels of blood Fe (median; 357 µg/mL vs. 485 µg/mL; *p* < 0.05), Mn (median; 6.78 ng/mL vs. 10.3 ng/mL; *p* < 0.05) and Se (median; 130 ng/mL vs. 151 ng/mL; *p* < 0.03) were lower than in HC males.

In OPP females, blood Cu increased compared with HC females (median; 1309 ng/mL vs. 982 ng/mL; *p* = 0.01). In OPP males, the contents of blood Cu (median; 1192 ng/mL vs. 858 ng/mL; *p* < 0.03) and Pb (median; 44.7 ng/mL vs. 17.9 ng/mL; *p* < 0.03) were higher with respect to HC males.

In NOPP males, the levels of blood Cr (median; 0.70 ng/mL vs. 0.35 ng/mL; *p* < 0.05), Cu (median; 1212 ng/mL vs. 858 ng/mL; *p* < 0.05) and Pb (median; 40.1 ng/mL vs. 17.9 ng/mL; *p* < 0.05) were higher, whilst blood Se was lower than the levels detected in HC males (median; 120 ng/mL vs. 151 ng/mL; *p* < 0.01).

Moreover, the whole blood levels of metals in PDAC patients with stage IV cancer were comparable with the levels in PDAC patients belonging to the other cancer stages.

Table 3 reports on the concentrations of toxic metals and essential elements determined in cancer and non-cancer tissues. Data showed that the levels of Cu (*p* < 0.01), Se (*p* < 0.01), Fe (*p* < 0.05) and Zn (*p* < 0.05) significantly increased in cancer tissue (ca. 2 to 4 times higher) than in the healthy tissue of the same patient. Moreover, some differences were observed when patients were stratified by gender. In males, the levels of Cu (median; 5045 ng/g vs. 690 ng/g; *p* < 0.01), Se (median; 732 ng/g vs. 96.6 ng/g; *p* < 0.01) and Zn (median; 49.8 ng/g vs. 7.68 ng/g; *p* < 0.01) resulted ca. 6 times higher in cancer tissues with respect to healthy tissues. In females, the concentrations of Se (median; 576 ng/g vs. 269 ng/g; *p* < 0.03) were ca. 2 times higher in cancer tissues with respect to healthy tissues.

As observed in the whole blood, there were no differences in the metal content in tissues depending on the PDAC stage.

Furthermore, ROC analysis was used as a diagnostic test to determine the metal concentrations able to discriminate PDAC patients from HC. In blood, the concentrations of 900 ng/mL of Cu (AUC, 0.769; sensitivity, 87%; specificity, 60%) and 0.35 ng/mL of Cr (AUC, 0.706; sensitivity, 84%; specificity, 60%) accurately divided patients with PDAC neoplasms from the HCs. In cancer tissues, the concentrations of 131 ng/g of Se (AUC, 0.816; sensitivity, 95.7%; specificity, 62%), 998 ng/g of Cu (AUC, 0762; sensitivity, 96%; specificity, 61.9%), 13.9 µg/g of Zn (AUC, 0.700; sensitivity, 96%; specificity, 61.9%) and 88.8 µg/g of Fe (AUC, 0.683; sensitivity, 91.3%; specificity, 57.1%) accurately discriminated PDAC tissues of patients with respect to healthy tissues in the same patients.

## 4. Discussion

PDAC is a highly aggressive lethal neoplasm considering the challenges of early diagnosis and due to poor response to treatments. Among all pancreatic cancers, PDAC represents more than 90% of all cases, and notwithstanding the scientific progress on this disease, PDAC has an average 5-year survival rate of less than 10% [19]. PDAC is considered to have a multifactorial origin, and among the possible risk factors, exposure to toxic metals or the imbalance of essential elements may alter normal metal homeostasis in the body, posing the way to activate molecular mechanisms involved in metal-induced malignant transformation or metal effects on tumor behavior [20].

Analysis of blood samples provides information on metal exposure at the long and medium terms. Metals are found in red blood cells (e.g., Cd, Mn and Pb), plasma components (Se), or occur unbound in blood [21]. Blood metal composition may reflect changes due to a diseased condition such as in PDAC, or it may reflect contributions of factors ranging from genetics to lifestyles. On the other hand, the analysis of healthy tissue adjacent to cancer tissues from the same individual served as an internal control, thus reducing individual-specific (i.e., age, gender, environment and genetics) and anatomical site-specific effects.

In this study, whole blood Cr was found to be significantly increased in PDAC patients (median, 0.70 ng/mL), OPPs (median, 0.70 ng/mL) and NOPPs (median, 0.64 ng/mL) with respect to the HCs (P50, 0.38 ng/mL). Cr(VI) compounds are known carcinogens, and are included in Group 1 (carcinogenic to humans) by the International Agency for Research on Cancer (IARC) classification [22]. Nevertheless, Cr ions may activate Fenton-like reactions, generate hydroxy radicals that induce oxidative stress, activate transcription factors such as nuclear factor kappa B (NF-κB), activate protein-1 (AP-1), tumor protein P53 (p53) and hypoxia-inducible factor-1 (HIF-1), regulate the cell cycle and induce apoptosis [23,24]. In previous literature, a study reported a high level of Cr in the serum of subjects with acute pancreatitis [25]. In addition, significantly increased levels of Cr in pancreatic juice were quantified in three population groups, and the levels of Cr followed a decreasing order: pancreatic cancer > pancreatitis > controls [20]. On the other hand, other authors did not find associations between self-reported regular exposure to Cr and the risk of developing PDAC [26], and between the levels of Cr in the toenails of patients with pancreatic cancer [27]. Similarly, a study reported comparable toenail levels of Cr between the controls and PDAC patients with a mutated and wild-type Kirsten rat sarcoma (KRAS) viral oncogene homolog [28]. In addition, similar findings, with no variations in the urinary Cr content of PDAC patients and controls, were also reported [29].

Regarding Cu, it was found to be significantly increased in the whole blood of PDAC patients (median, 1251 ng/mL), OPPs (median, 1274 ng/mL) and NOPPs (median, 1148 ng/mL) with respect to the HCs (median, 982 ng/mL), and this result was confirmed in PDAC, OPP and NOPP males and females with respect to HC males and females. Significantly higher Cu levels were also observed in pancreatic cancer tissue (median, 4664 ng/g) with respect to the adjacent healthy tissue (median, 1330 ng/g), and the same significant finding was observed in PDAC males, but not in PDAC females. Copper is an integral part of Cu-based metalloenzymes (as cytochrome oxidase, NADH dehydrogenase-2, Cu/Zn superoxide dismutase) and has antioxidant capacity, lowering oxidative stress as well as stabilizing cellular functions. Therefore, when Cu homeostasis is not regulated, Cu accumulation may create the risk of cellular injuries [25]. Moreover, in cancer and inflammation, plasma Cu and ceruloplasmin concentrations were raised, and the rates of synthesis and secretion of ceruloplasmin by the liver were enhanced. The elevated ceruloplasmin concentrations in both conditions would provide additional Cu uptake by cells in normal tissues and cancer cells [30]. In addition, Cu-dependent amino oxidases may mediate angiogenesis that plays a critical role in the growth of cancer [30]. In the literature, two different studies observed elevated levels of Cu in the serum of pancreatic patients with respect to the controls [14,30]. In addition, a higher serum Cu content in patients with acute pancreatitis and higher urinary Cu levels in PDAC patients were observed [26,29]. Furthermore, Cu in pancreatic juice was 2 times higher with respect to the Cu levels detected in acute pancreatitis, but comparable to Cu contents in the controls [20]. Opposite findings were found by other authors that reported no differences in Cu concentrations in the toenails of PDAC patients and the controls [27,28].

Although Fe is essential for the function of many cellular enzymes, an excess of Fe may result in the production of ROS that can lead to peroxidation and apoptosis by attacking protein, lipids, nucleic acids and carbohydrates, processes closely related to tumorigenesis [9,31]. In this study, Fe levels in whole blood were decreased in male PDAC patients with respect to HC males. Contrarily, Fe concentrations in PDAC tissue (median, 173 µg/g) were elevated compared to healthy tissue (median, 94.8 µg/g). A growing body of evidence suggests that an Fe imbalance can be a key factor in pancreatic cancer. In general, the dependence of cancer cells on Fe uptake is fully recognized because this element is responsible for cell respiration, oxygen transport, oxygen metabolism, energy metabolism, DNA synthesis, etc. In particular, the incremented Fe level in cancer cells was linked to tumor-associated angiogenesis, which is the development of new blood vessels in response to proteins secreted by tumor cells [32,33]. Other studies suggested that the Fe overload in pancreatic islets impaired insulin secretion and β-cell function, and accelerated pancreatic β-cell death [34]. In addition, exposure to an excess of Fe may induce epithelial–mesenchymal transition (EMT) in pancreatic cancer cells, loss of p53 and suppression of p53 transcriptional activity [13]. Higher serum Fe was found to be associated with pancreatic cancer, although not consistently [35,36,37]. Authors reported higher levels of serum ferritin in pancreatic cancer, and Fe homeostasis genes and ferroptosis genes significantly altered with pancreatic tumor grades [9]. Epidemiological studies have suggested positive associations between Fe and red meat intake, and the risk of PDAC [38].

Although the IARC did not consider Pb to show a clear carcinogenic activity (Group 2A, probably carcinogenic to humans), this metal is extensively distributed in the environment and may cause numerous acute and chronic circulatory, neurological, hematological, gastrointestinal, reproductive and immunological pathologies [39]. Lead can inhibit delta-aminolevulinic acid dehydratase (δ-ALAD), disrupting heme synthesis in bone marrow erythroblasts. In addition, it can induce oxidative stress interfering with antioxidant activities by inhibiting functional SH groups in several enzymes such as superoxide dismutase (SOD), catalase (CAT), glutathione peroxidase (GPx), glucose-6-phosphate dehydrogenase (G6PD) and δ-ALAD [40]. In this study, Pb levels were comparable between diseased patients and the controls upon both whole blood and tissue analyses. On the other hand, differences in whole blood Pb levels were found as a function of gender. Indeed, blood Pb was significantly (ca. 2.5 times) higher in PDAC males with respect to PDAC females. The reason for this result is that males have more erythrocytes (ca. 10%) than females and there are different long-term Pb kinetics in men and women. In fact, Pb is stored in bone for 10–30 years, which hold approximately 90% of the body burden and, during the life span, it is released back into the blood (as an “endogenous source”) via bone remodeling [41,42]. There is no conclusive evidence for the Fe involvement in PDAC neoplasms. Some authors found unchanged levels of Pb between PDAC patients and the controls in different matrices, such as urine [29], pancreatic juice [20] and blood [43]. On the other hand, significantly elevated levels of Pb in the toenails of pancreatic cancer patients [27] and significantly decreased serum levels of Pb in pancreatic cancer patients [14] were also observed. Another study showed that higher levels of Pb may be a risk factor for both KRAS-mutated and wild-type cases of pancreatic cancer [28].

Regarding Se, it is a micronutrient required for the proper functioning of the body and takes part in several anti-carcinogenic mechanisms, including inactivating oxygen free radicals and initiating major DNA repair pathways [12]. Furthermore, selenoproteins and Se metabolites reduced pancreatic carcinogenesis in an animal model [44]. Considering this study, no differences were noted in Se whole blood levels among the patients and controls, but some relevant results were observed according to gender. In particular, Se levels in the whole blood of PDAC males and NOPP males were significantly decreased with respect to Se content in the whole blood of HC males. On the other hand, Se levels in cancer tissues (median, 598 ng/g) were significantly higher than in the corresponding healthy tissues (median, 223 ng/g). Higher Se tissue concentrations were found in both females and males with respect to the adjacent healthy tissues. Regarding Se and PDAC, there are some controversial findings. Two studies found higher levels of Se in patients with pancreatic cancer [20,30]. Contrastingly, other investigations reported that high concentrations of Se or a high intake of Se might reduce the risk of pancreatic cancer [12,27]. Contrarily, other authors did not find associations between Se and pancreatic cancer risk [44].

There is a lot of information involving Zn dysregulation in the development of pancreatic cancer. For example, the overexpression of Zip4 protein was reported in 94% of clinical PDAC tissue compared with adjacent normal tissue, and malignant cells displayed significantly higher Zip4 expression compared with normal pancreatic cells [16,45]. Further, whereas Zip4 expression may be increased in some cancerous tissues, Zip3 and Zip8 were reported as down-regulated in some adenocarcinomas [17]. Decreased serum Zn levels were associated with chronic pancreatitis, and this disease was linked with lower levels of Cu/Zn-SOD and metallothionein (MT), responsible for the increase in ROS and elevation in oxidative stress [15]. In the present study, the levels of Zn in the whole blood were unchanged between PDAC patients and the controls. In PDAC, tissue concentrations of Zn were significantly elevated with respect to the levels in healthy tissues (median, 43.7 µg/g vs. 24.1 µg/g). This result was confirmed when the cancer and non-cancer tissue of males were compared. The present findings were consistent with those reported in a previous study, where the Zn content in tumor tissues was higher than that in adjacent non-cancer tissues, also indicating that the high concentration of Zn was beneficial for the occurrence and development of pancreatic cancer [46]. In addition, an increased level of Zn was found in the urine of PDAC patients [29]. These authors postulated that oxidative stress caused by cancer development and progression oxidizes the sulfhydryl groups in cysteine, decreasing the Zn-binding capacity with a consequent higher amount of free Zn that is rapidly eliminated through urine. Conflictingly, other authors reported decreased levels of Zn in subjects with acute pancreatitis and pancreatic cancer, or in rats with induced pancreatitis [14,25,43]. In addition, it was observed that the Zn concentration in pancreatic fluid was comparable in patients with chronic pancreatitis and pancreatic cancer compared to a normal pancreas [47].

The use of trace element ratios instead of single trace element content was previously reported in studies, for example, in lung cancer [48], open-angle glaucoma patients [49] and idiopathic pulmonary fibrosis [50], in order to reflect the dyshomeostasis of metals associated with disorders. Thus, in this study, the ratio between metals was investigated as a possible indicator of PDAC disease. In particular, the whole blood Cu/Zn ratio in PDAC (0.185) patients was found to be ca. 1.3-fold higher than in the controls (0.143), resulting in a statistically different level at *p* < 0.01. The disruption of the whole blood Cu/Zn ratio may be responsible for the increased oxidative stress and decreased blood antioxidant capacity in PDAC patients. This ratio was found to be increased in cases of acute pancreatitis in patients [25].

## 5. Conclusions

Environmental exposure to chemicals, genetics and lifestyle are among the possible risk factors of PDAC development and progression. Among these factors, exposure to toxic metals or the imbalance in essential elements may have an impact on PDAC neoplasm. The results of the present study suggest that whole blood (Cr and Cu) and tissue (Cu, Fe, Se, and Zn) levels of toxic metals and essential elements in PDAC patients were different from those observed in the healthy controls. These metals may have a role in PDAC-associated metabolic pathways, including the increase in ROS production, boosting lipid peroxidation, depleting of antioxidant barriers, enhancing the expression of inflammation genes, modifying DNA bases, etc. Moreover, the higher Cu/Zn ratio may indicate an increased level of oxidative stress and decreased antioxidant properties in PDAC neoplasms. Although studies with a high number of PDAC patients are required to support the present findings and better understand the underlying pathophysiological processes, the levels of metals in blood and cancer tissue can be utilized as biomarkers of PDAC diagnosis and monitoring.

## Figures and Tables

**Table 1:** Descriptive characteristics of PDAC patients and controls.

	PDAC	Controls
Subject (no.)		20
Total population	46	
Operated PDAC patients (OPPs)	32	
Non-operated PDAC patients (NOPPs)	14	
Mean age (years)	67.2 ± 9.4	60.5 ± 8.5
Sex (no.)		
Females	25	11
Males	21	9
Cancer stages (no.)		
Stage 0	5	
Stage Ia	6	
Stage Ib	5	
Stage IIb	10	
Stage III	6	
Stage IV	14	
Metastasis (no.)		
Yes	14	
No	32	

**Table 2:** Concentration of toxic metals and essential elements (5th–50th–95th percentiles) in the whole blood of PDAC patients and controls.

	PDAC Patients (No. 46)	OPPs (No. 32)	NOPPs (No. 14)	HCs (No. 20)	Statistical Test (Patients vs. HCs)
Cd (ng/mL)	0.21–0.58–1.52	0.27–0.58–1.51	0.20–0.54–1.47	0.11–0.50–1.57	ns
Cr (ng/mL)	0.26–0.70–1.63 ^a^	0.30–0.74–1.60 ^b^	0.26–0.64–1.59 ^b^	0.17–0.38–1.33	^a^ *p* < 0.01; ^b^ *p* < 0.05
Cu (ng/mL)	853–1251–1682 ^a^	914–1274–1775 ^a^	851–1148–1670 ^b^	779–982–1312	^a^ *p* < 0.01; ^b^ *p* < 0.05
Fe (µg/mL)	234–400–608	236–398–588	240–407–610	324–426–689	ns
Mn (ng/mL)	3.79–7.24–15.8	3.78–7.24–14.6	4.13–7.43–15.8	5.63–9.81–16.4	ns
Ni (ng/mL)	0.44–0.83–2.36	0.44–0.79–2.54	0.47–0.84–1.77	0.41–0.77–2.02	ns
Pb (ng/mL)	8.48–29.6–94.3	8.38–23.4–53.4	9.43–32.2–112	6.76–16.8–63.5	ns
Se (ng/mL)	90.6–129–203	101–149–233	90.4–119–176	108–151–187	ns
Zn (µg/mL)	4.04–6.86–10.0	4.71–7.99–10.6	3.93–6.41–9.42	5.31–7.02–10.2	ns

OPPs = Operated PDAC patients; NOPPs = Non-operated PDAC patients; HCs = Healthy controls; ns: Not significant, a = patients vs. HCs significant at *p* value less than 0.01, b = patients vs. HCs significant at *p* value less than 0.05.

**Table 3:** Concentration of toxic metals and essential elements (5th–50th–95th percentiles) in cancer and non-cancer tissue collected from the same PDAC patients.

	Cancer Tissue (No. 32)	Non-Cancer Tissue (No. 32)	Statistical Test (Cancer vs. Non-Cancer)
Cd (ng/g)	84.2–1593–5080	43.8–346–6883	ns
Cr (ng/g)	7.90–31.3–590	12.2–32.6–309	ns
Cu (ng/g)	2232–4664–6307	485–1330–5557	*p* < 0.01
Fe (µg/g)	60.4–172–552	47.9–94.8–319	*p* < 0.05
Mn (ng/g)	214–1098–3532	25.3–296–3891	ns
Ni (ng/g)	18.0–89.7–1919	14.3–56.5–1806	ns
Pb (ng/g)	6.33–23.4–130	4.44–12.1–73.4	ns
Se (ng/g)	327–598–1350	40.2–223–738	*p* < 0.01
Zn (µg/g)	27.3–49.7–107	3.78–24.1–116	*p* < 0.05

ns: Not significant.

## Data Availability

Data are unavailable due to privacy or ethical restrictions.

## Supplementary Materials

The following supporting information can be downloaded at: https://www.mdpi.com/article/10.3390/toxics12010032/s1. “Methodology for concentration of toxic metals and essential elements in blood and tissues of patients with Pancreatic Ductal Adenocarcinoma”; “Table S1: ICP-MS instrumental characteristics and settings”; “Table S2: Results of the CRM ClinChek^®^ Whole Blood Control, Level I (Recipe, Munich, Germany) analysis”; “Table S3: Results of the CRM pig kidney ERM-BB186 (IRMM, Geel, Belgium) analysis”; “Table S4: Results of the CRM mussel tissue ERM-CE278k (IRMM, Geel Belgium) analysis”.
