# Supplementary material for: Toxic Metal and Essential Element Concentrations in the Blood and Tissues of Pancreatic Ductal Adenocarcinoma Patients
Source: Toxics. 2024 Jan 1;12(1):32. doi: 10.3390/toxics12010032 (PMC10818929; doi:10.3390/toxics12010032)
Supplement: Supplementary file 1 [file toxics-12-00032-s001.zip › toxics-2756311-supplementary.pdf]

SUPPLEMENTARY MATERIALS

Methodologies for concentration of toxic metals and essential elements in blood and tissues of patients with Pancreatic Ductal Adenocarcinoma

Quantification of Cd, Cr, Cu, Fe, Mn, Ni, Se, Pb and Zn in whole blood and tissue was performed by iCAP Qc ICP-MS (Inductively Coupled Plasma-Mass Spectrometry). To reduce polyatomic interferences on analytical masses, the He pressurized QCell in Kinetic Energy Discrimination (KED) mode was used for the quantification of elements. ICP-MS instrumental characteristics and settings are reported in Table S1. Quantification was performed by the addition calibration method, and multi-calibration curves were prepared using pooled digested samples and mono-elemental reference standards (CPAChem, Bogomilovo, Bulgaria). Rhodium as internal standard was added at 1 ng/ml in the analytical solutions to account for possible instrumental drifts, and certified reference materials (CRMs) (i.e., ClinChek® Whole Blood Control Level I, pig kidney ERM-BB186, and mussel tissue ERM-CE278k) were analysed to evaluate method recovery and precision (Tables S2-S4). Method limits of detection (LoDs) were calculated as three time the standard deviation of replicated measurements of pooled digested samples. The following LoDs were obtained in whole blood: Cd, 0.03 ng/ml; Co, 0.02 ng/ml; Cr, 0.05 ng/ml; Cu, 100 ng/ml; Fe, 10 µg/ml; Mn, 0.4 ng/ml; Ni, 0.1 ng/ml; Pb, 0.5 ng/ml; Se, 10 ng/ml; Zn, 0.15 µg/ml; whilst, LoDs in tissues were as follows: Cd, 3 ng/g; Co, 0.1 ng/g; Cr, 1 ng/g; Cu, 40 ng/g; Fe, 2.5 µg/g; Mn, 3 ng/g; Ni, 2.5 ng/g; Pb, 1 ng/g; Se, 3 ng/g; Zn, 0.3 µg/g. For both matrices, procedural blanks were analysed to control for possible exogenous metals contamination from plastics and reagents.

Table S1. ICP-MS instrumental characteristics and settings.

**Spectrometer**	iCAP Qc ICP-MS (Thermo Scientific, Bremen, Germany)
**Rf power**	1550 W
**Interface**	Sampler and skimmer cones in Ni
**Nebulizer**	Cross flow
**Expansion chamber**	Cyclonic
**Argon flow**	Plasma, 14 L/min; Nebulizer, 1.07 L/min; Auxiliary, 0.8 L/min
**Data acquisition**	3 runs, 15 passes
**Extraction lens**	-76.4 V
**CCT Focus lens**	-3.72 V
**Focus lens**	-8.50 V
**Analyzing mode**	Kinetic Energy Discrimination (KED)
**Collisional He flow**	4 ml/min
**Analytical masses**	^{114}Cd, ^{52}Cr, ^{63}Cu, ^{56}Fe, ^{55}Mn, ^{60}Ni, ^{82}Se, ^{208}Pb and ^{64}Zn, all in KED mode
**Internal standard**	^{103}Rh

Table S2. Results of CRM ClinChek® Whole Blood Control, Level I (Recipe, Munich, Germany) analysis.

**Elements**	**Certified (Range)**	**Found (%)**
Cd (ng/ml)	1.58 (1.18-1.97)	1.42 (90)
Cr(ng/ml)	2.23 (1.67-2.78)	2.12 (95)
Cu (ng/ml)	738 (590-885)	790 (107)
Fe (µg/ml)	345 (276-414)	352 (102)
Mn (ng/ml)	7.90 (6.32-9.48)	7.27 (92)
Ni (ng/ml)	2.10 (1.58-2.63)	2.31 (110)
Pb (ng/ml)	37.6 (30.1-45.2)	39.1 (104)
Se (ng/ml)	83.1 (66.5-99.7)	80.6 (97)
Zn (ng/ml)	4430 (3550-5320)	4784 (108)

Table S3. Results of CRM pig kidney ERM-BB186 (IRMM, Geel, Belgium) analysis.

Elements	Certified ± Uncertainty	Found (%)
Cd (mg/kg)	1.09 ± 0.05	1.06 (97)
Cu (mg/kg)	36.5 ± 1.8	37.6 (103)
Fe (mg/kg)	255 ± 13	271 (106)
Mn (mg/kg)	7.26 ± 0.25	6.97 (96)
Pb (mg/kg)	0.040 ± 0.005	0.037 (94)
Se (mg/kg)	10.3 ± 0.9	11.5 (112)
Zn (mg/kg)	134 ± 5	132 (99)

Table S4. Results of CRM mussel tissue ERM-CE278k (IRMM, Geel Belgium) analysis.

**Elements**	**Certified ± Uncertainty**	**Found (%)**
Cd (mg/kg)	0.336 ± 0.025	0.342 (102)
Cr (mg/kg)	0.730 ± 0.22	0.715 (98)
Cu (mg/kg)	5.98 ± 0.27	5.74 (96)
Fe (mg/kg)	161 ± 8	156 (97)
Mn (mg/kg)	4.88 ± 0.24	5.27 (108)
Ni (mg/kg)	0.69 ± 0.15	0.81 (117)
Pb (mg/kg)	2.18 ± 0.18	2.41 (107)
Se (mg/kg)	1.62 ± 0.12	2.11 (97)
Zn (mg/kg)	71 ± 4	73 (103)
